# Late blight field resistance in potatoes carrying *Solanum americanum* resistance genes (Rpi-amr3 and Rpi-amr1)

**DOI:** 10.1080/21645698.2025.2479913

**Published:** 2025-03-23

**Authors:** Svante Resjö, Iqra, Nam P Kieu, Muhammad Awais Zahid, Marit Lenman, Björn Andersson, Erik Andreasson

**Affiliations:** a Department of Plant Protection Biology, Swedish University of Agricultural Sciences, Lomma, Sweden; b Department of Forest Mycology and Plant Pathology, Swedish University of Agricultural Sciences, Uppsala, Sweden

**Keywords:** GM field trial, late blight, *Phytophthora infestans*, resistance gene, Rpi-amr1, Rpi-amr3, *Solanum americanum*

## Abstract

Potato (*Solanum tuberosum* L.) is an important global crop, but its production is severely impacted by late blight, caused by the pathogen *Phytophthora infestans*. The economic burden of this disease is significant, and current control strategies rely mainly on fungicides, which face increasing regulatory and environmental constraints. To address this challenge, potatoes with resistance genes from wild potato relatives offer a promising solution. This study evaluated field resistance to late blight in potato lines (Maris Piper) containing the *Solanum americanum* resistance genes *Rpi-amr3* and *Rpi-amr1* across three years (2018–2020) in Sweden. Field trials were conducted under natural infection conditions to assess disease resistance. Results showed that the transgenic lines conferred strong resistance to late blight compared to the susceptible control. However, slight late blight symptoms were observed in the transgenic lines. These results highlight the effectiveness of *S. americanum* resistance genes in providing strong resistance, and emphasize the potential of stacking multiple R genes, including these genes to maintain efficacy. This research supports the development of resistant potato varieties as a sustainable alternative to chemical control, promoting food security and environmentally friendly agriculture.

## Introduction

Potato (*Solanum tuberosum* L.) ranks as one of the most important globally cultivated crops, with an annual production of 370 million tonnes in 2019.^1^ It is the third most consumed crop worldwide, highly valued for its rich nutritional content, including carbohydrates, proteins, minerals, and vitamins (Naumann et al., 30). However, potato production faces significant challenges from plant diseases, such as late blight, caused by the oomycete pathogen *Phytophthora infestans*. This disease poses a major threat to potato production and causes annual losses with a global economic impact estimated to €6.1 billion^2,3^ and is also a major problem in Sweden.^4,5^ Under favorable conditions with moderate-to-high humidity and moderate temperatures, an unprotected potato field with a susceptible cultivar can be severely damaged by *P. infestans* in just a few days^6^

Conventional breeding to pyramid resistance genes in potato is complicated because efficient back crossing is practically impossible. In contrast, genetic modification (GM) technology offers a more rapid approach offering a significant and sustainable reduction in the need for fungicides to control late blight.^7,8^ Some wild *Solanum* species have demonstrated high resistance to many potato diseases, especially late blight, and have been integral to breeding programs for many years.^9^ Several resistance genes against *P. infestans* (*Rpi)* genes have been characterized across different *Solanum* species, including R2 (*Rpi-blb3*), *Rpi-vnt1*, *R3a, R8*, and *Rpi-blb2*,^10–14^ but some of these are not any longer functional in agriculture due to their very narrow specificity and changed *Phytophthora* populations. One reason for this could the fact that have been deployed as single genes. In order to be able to pyramid different resistance genes, it is necessary to have many different genes to choose from. *Solanum americanum*, a wild species closely related to a common weed in Europe *Solanum nigrum*, has shown a genetic potential for strong resistance against *P. infestans*^15–17^ Previously, the relatively broad resistance genes *Rpi-amr1* and *Rpi-amr3* have been cloned from various accessions of *S. americanum*,^15,17,18^ and *Rpi-amr3* have been shown to be efficient to control late blight in GM potato field trials in England.^18^

In this study, we conducted multi-year field trials in Sweden with only natural infection to assess the effectiveness of *Solanum americanum*-derived resistance genes *Rpi-amr1* and *Rpi-amr3* in potatoes. These trials aimed to evaluate the resistance of potential NGT/GM potatoes to late blight. Field tests with *Rpi-amr1* has never been reported before. Southern Sweden is a good location for these experiments due to conducive environmental conditions for stable late blight infections caused by diverse pathogen populations.

## Materials and Methods

### Plant Material

The genetically modified potatoes used in this experiment were derived from the cultivar Maris Piper. Genetic transformations were performed using *Agrobacterium tumefaciens* to introduce resistance genes, *Rpi-amr3* or *Rpi-amr1*, from *Solanum americanum*. Three single R-gene transformed lines, designated as Rpi-amr3_5C, Rpi-amr1D_15A (allele 1101), and Rpi-amr1D_15B (allele 1101), were selected for testing.^15,17^ These were compared to a non-transformed Maris Piper line, which served as the susceptible control treatment. All lines were maintained under the same *in vitro* conditions.^19^

### Detach Leaf Assay

*P*. *infestans* strain 88,069 was cultivated on solid rye sucrose medium, in Petri dishes incubated at 18°C in darkness, and sub-cultured every three to four weeks. Sporangia were harvested by flooding 14-day-old cultures with cold (4°C) deionized water and gentle rubbing. The resulting suspensions were filtered through 40 μm nylon mesh and concentrated to 50 000 sporangia/mL. Twenty-five µL of the spore solution was pipetted, and the leaves were maintained in a humid environment (RH ~ 100%) under controlled conditions.^20^ Results were recorded by measuring the infection size of each leaflet at 7 days post inoculation (dpi).

### Field Trials

The field trials were conducted over three consecutive years (2018, 2019, and 2020) with natural infection conditions at an established field trial site with a four-year crop rotation in the Borgeby research farm in southern Sweden (geographic coordinates: 55.75289, 13.04872). The experimental setup included three transgenic lines and one control line, arranged in a randomized complete block design with four replications, except for year 2018 with only two replicates were used. Each replication consisted of a row of ten plants. To minimize virus interference, the trial was sprayed with mineral oil.^21^ Planting dates were 7 of June 2018, 21 of May 2019 and 27 0f May 2020.

The late blight susceptible cultivar Bintje was planted around the perimeter of the trial field and left untreated to ensure an even infection pressure. The experiments were conducted under a permit approved by the Swedish Board of Agriculture (Dnr 4.6.18–10775/16), in compliance with the regulations outlined in the “Environmental Code” (1998:808), the Code of Regulations of the Swedish Board of Agriculture (SJVFS 2003:5) related to transport and labeling, and Regulation 2002:1086 regarding the deliberate release of GMOs into the environment.

At the end of the season of 2020, tubers from each block were harvested and a number of tubers were recorded. The tuber yield was measured in kilogram per meter square for each line and then transformed to tons per hectare. The percent increase in yield of transgenic was calculated by using following formula:

$$\mathrm{Percentage}\;\mathrm{increase}\;\mathrm{in}\;\mathrm{yield}\;\;\;\;\;\;\;\;\;\;\;\;\;\;\;\;\;\;\;\;\;\;\;\;\;\;\;\;\;\;\;\;\;\;\;\;\;\;\;\;\;\;\;\;\text{\textbackslash{}break\textbackslash{}break}=\frac{\mathrm{Yield}\;\mathrm{of}\;\mathrm{control}\;\mathrm{line}-\mathrm{Yied}\;\mathrm{of}\;\mathrm{transgenic}\;\mathrm{line}}{\mathrm{Yield}\;\mathrm{of}\;\mathrm{transgenic}\;\mathrm{line}}*100.$$

### Scoring of Late Blight Disease in Field

The severity of late blight disease was visually assessed throughout the growing season by estimating the percentage of infected leaf area twice a week as described in the guidelines for field trials of the Swedish University of Agricultural Sciences (Swedish University of Agricultural Sciences 2011). The percentage of infection was rated from 0 to 100, where 0 indicated no disease symptoms and 100 represented complete plant death with no green leaves remaining. Disease scoring was done from the time of first late blight symptoms on the control plants and was conducted twice a week until the end of the growing season. The disease incidence of late blight based on area under disease progression curve (AUDPC) was also calculated.^22^

### Pathogen Population

Pathogen sampling was done by using FTA-cards.^31^ For each sample, a leaflet having only one lesion was pressed onto the sampling area of the FTA card with the sporulating side facing down, and plant residues were subsequently removed. The FTA cards were dried and kept in room temperature before being sent to the James Hutton Institute for genotyping. Then genotyping was done by using microsatellite DNA fingerprinting with a 12-plex Simple Sequence Repeats (SSRs) method.^32^ The SSR genotype data was analyzed by the Minimum Spanning Network clustering method^33^ using the Bruvo’s distance function^23^ in the Poppr package v.2.9.3. in R.

### Statistical Analysis

The disease and yield data for each year were analyzed by using pairwise Student′s t-test for comparing genetically modified line with wild type control (Maris Piper). The data on disease severity over time for the different lines were analyzed using one-way Anova in R.

## Results

Late blight resistance in the potato lines carrying *Rpi-amr3* and *Rpi-amr1* in Maris Piper background were confirmed by detached leaf assays. No symptoms could be detected in artificial inoculation with *P*. *infestans* model strain 88069, whereas the untransformed Maris Piper shown full susceptibly (whole leaf blighted after 7 days). Three-year field trial data (2018, 2019 and 2020) were collected to assess the field resistance of *Rpi-amr3* and *Rpi-amr1* to natural infestation of late blight in Southern Sweden.

### *Late Blight Scoring and* Phytophthora *Population 2018*

**Figure 1.**
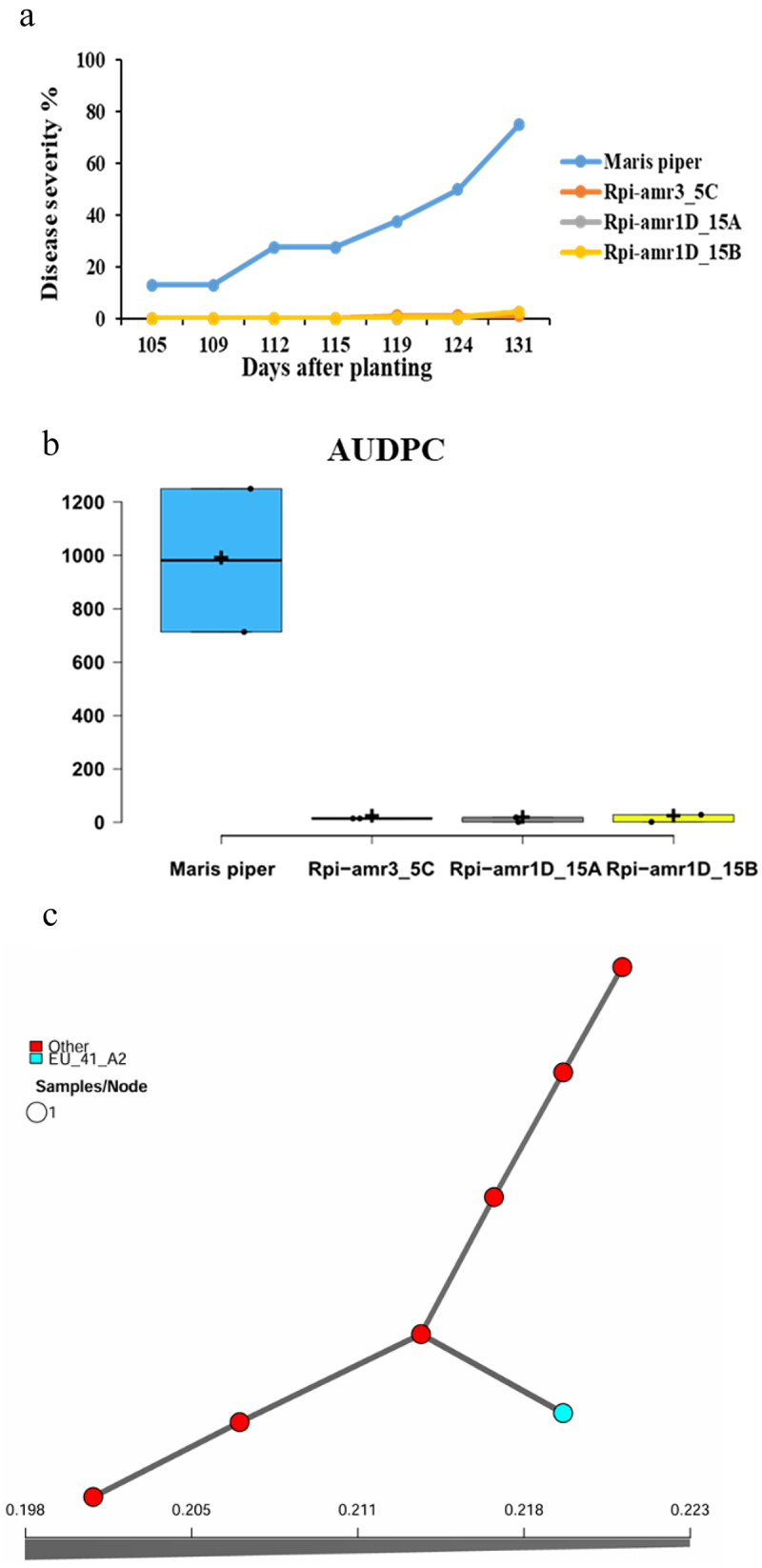
(a) Visual assessment of transgenic lines (Rpi-amr3_5C, Rpi-amr1D_15A, Rpi-amr1D_15B) and Maris Piper (control) with late blight of potato. The x-axis indicates the days after planting. The recordings for the transgenic lines Rpi-amr3_5C, Rpi-amr1D_15A and Rpi-amr1D_15B are overlapping in the graph. The y-axis indicates the disease severity of late blight symptoms in percentage. (b) The area under disease progress curve (AUDPC) is shown for each genotype in field trial year 2018. The x-axis indicates the tested genotypes in three consecutive year. The y-axis indicates the mean values for disease progression curve. *N* = 2. (c) Minimum spanning network (MSN) of *P. infestans* using Bruvo’s distance for samples collected in 2018 in this field. MSN is based on 12 SSR markers and each circle represents a unique multilocus genotype. Branch thickness represents genetic relatedness. Circle size displays the number of samples of each MLG. Circles in red represent unidentified genotypes, while the circle in blue represents a genotype previously characterized by the Euroblight network (www.Euroblight.net).

The field experiments indicate that during 2018, all three the transgenic lines, Rpi-amr3_5C, Rpi-amr1D_15A and Rpi-amr1D_15B conferred resistance to late blight of potato under field conditions, whereas the wild type Maris piper (control treatment) was completely infected within five weeks after the first appearance of symptoms (Figure 1a). Mean values of AUDPC of each genetically modified lines were compared with control treatment (as two replications of each line in year 2018) and it implies that *Rpi-*amr3 and *Rpi-amr1D* reduce infection of late blight of potato under field conditions (Figure 1b).

During 2018, only seven samples of *P. infestans* were collected and genotyped. Of these, only one had been recorded earlier as SSR multilocus genotype EU_41_A2. The other, earlier unidentified genotypes were highly diverse among each other and clearly distinct from EU_41_A2 (Figure 1c).

### *Late Blight Scoring and* Phytophthora *Population 2019*

**Figure 2.**
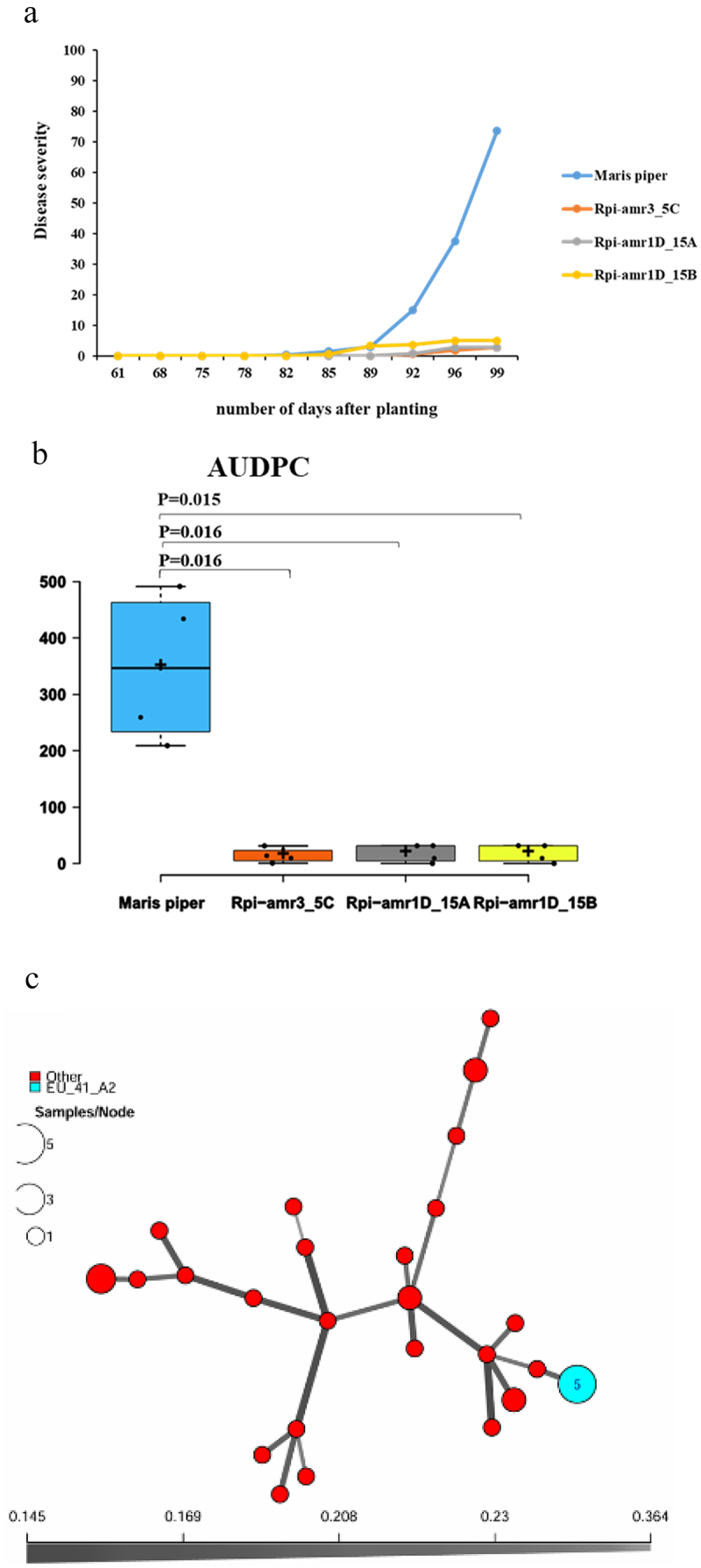
(a) Visual assessment of transgenic lines (Rpi-amr3_5C, Rpi-amr1D_15A, Rpi-amr1D_15B) and Maris Piper (control) to late blight of potato. The x-axis indicates the days after planting. The y-axis indicates the disease severity of late blight symptoms in percentage. (b) The area under disease progress curve (AUDPC) is shown for each genotype in field trial year 2019. The x-axis indicates the tested genotypes (transgenic line and control). The y-axis indicates the values of AUDPC. The *p* values are given for each transgenic line as compared to Maris Piper (control) in two-tailed student’s T-test. *N* = 4. (c) Minimum spanning network (MSN) of *P. infestans* using Bruvo’s distance for samples collected in 2019. MSN is based on 12 SSR markers and each circle represents a unique multilocus genotype (MLG). Branch thickness represents genetic relatedness. Circle size displays the number of samples of each MLG. Circles in red represent unidentified genotypes, while the circle in blue represents a genotype previously characterized by the Euroblight network (www.Euroblight.net).

The field trials 2019 indicate that all three transgenic lines Rpi-amr3_5C, Rpi-amr1D_15A, and Rpi-amr1D_15B – exhibited significant resistance to late blight in potatoes compared to the wild-type Maris Piper, which served as the control (Figure 2a). Additionally, the statistically significant lower values of the disease progression curve for all three genetically modified lines (Rpi-amr3_5C, Rpi-amr1D_15A, and Rpi-amr1D_15B) compared to control Maris Piper showed that these transgenic lines can effectively control the infection of late blight in the field (Figure 2b). In 2019, the disease score reached a maximum of 4%. During 2019, 34 samples of *P. infestans* were collected and genotyped. The analyzed samples consisted of earlier unidentified genotypes together with the EU_41_A2 genotype. Like in 2018, the samples displayed a high diversity among each other (Figure 2c).

### *Late Blight Scoring and* Phytophthora *Population 2020*

**Figure 3.**
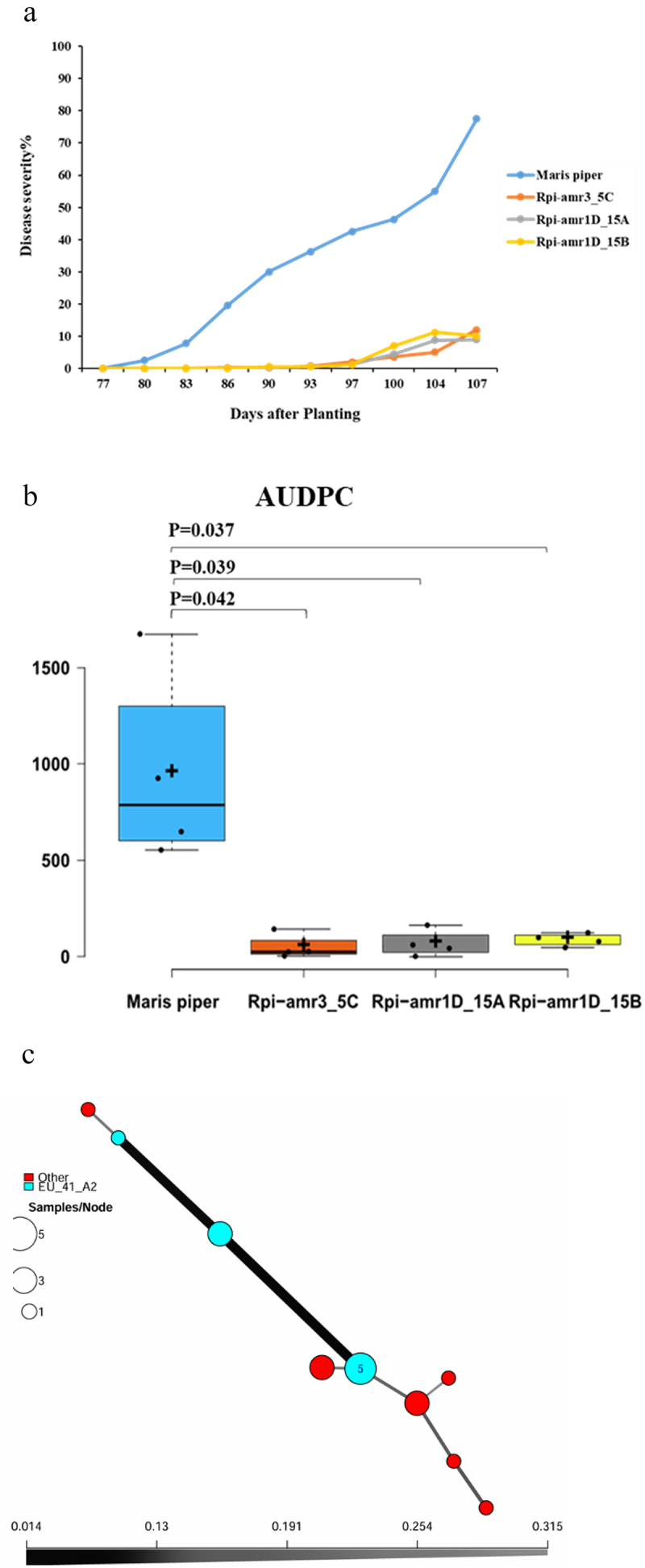
(a) Visual assessment of transgenic lines (Rpi-amr3_5C, Rpi-amr1D_15A, Rpi-amr1D_15B) and Maris Piper (control) to late blight of potato. The x-axis indicates the days after planting. The y-axis indicates the disease severity of late blight symptoms in percentage. (b) The area under disease progress curve (AUDPC) is shown for each genotype in field trial year 2020. The x-axis indicates all three transgenic line and control. The y-axis indicates the value of AUDPC. The *p* values are given for each transgenic line as compared to Maris Piper (control) in two-tailed student’s T-test. *N* = 4. (c). Minimum spanning network (MSN) of *P. infestans* using Bruvo’s distance for samples collected in Southern Sweden in 2020. MSN is based on 12 SSR markers and each circle represents a unique multilocus genotype (MLG). Branch thickness represents genetic relatedness. Circle size displays the number of samples of each MLG. Circles in red represent unidentified genotypes, while the circle in blue represents a genotype previously characterized by the Euroblight network (www.Euroblight.net).

The 2020 field experiment demonstrated that each transgenic genotype (Rpi-amr3_5C, Rpi-amr1D_15A, Rpi-amr1D_15B) had significantly lower AUDPC values and reduced disease severity in the disease scoring curve compared to Maris Piper. This suggests that *Rpi-amr3* and *Rpi-amr1* effectively prevent late blight infection in potatoes under field conditions (Figure 3a,b). In order to compare the disease severity in different years for each line, we performed a one-way Anova. In this analysis, disease severity did not vary significantly over time for any of the lines (Adjusted p-value (Benjamini-Hochberg): Maris Piper 0.20, Rpi.amr3i_5C 0.48, Rpi.amr7D_15A 0.38 and Rpi.amr7D_15B 0.20). Nineteen samples from 2020 were analyzed, and the results were consistent with the other years with a mix of “unknown” genotypes and EU_41_A2. A slight genetic differentiation within EU_41 _A2 could be observed (Figure 3c).

### Assessment of Yield and Number of Tubers

**Figure 4.**
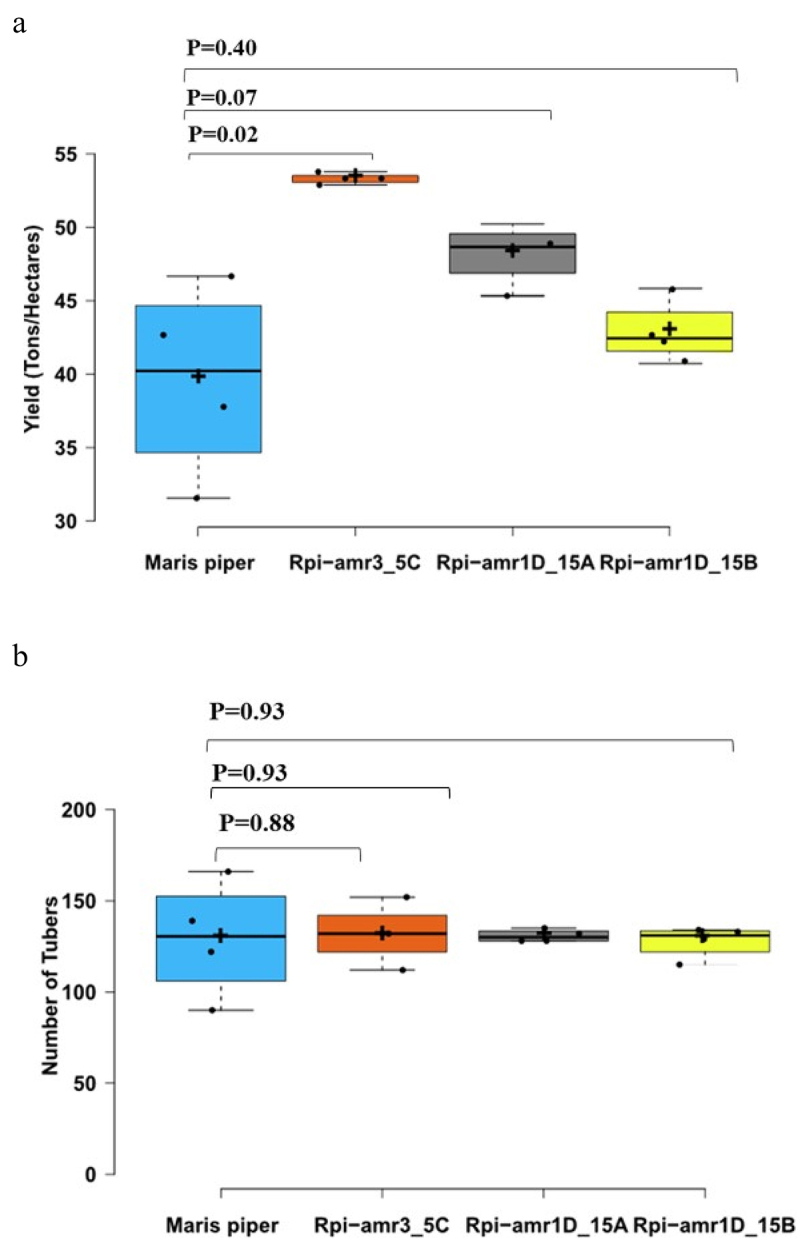
Yield and number of tubers. (a) The x-axis indicates yield in tons per hectare. The y-axis indicates all three transgenic line (Rpi-amr3_5C, Rpi-amr1D_15A, Rpi-amr1D_15B) and Maris Piper (control line). The *p* values are given for each trangenic line as compared to Maris Piper (control) in two-tailed student’s T-test. *N* = 4. (b) the number of tubers harvested for all three trangenic lines (Rpi-amr3-_5C, Rrpi-amr1D_15A, Rpi-amr1D_15B) and Maris Piper (control line). The *p* values are given for each transgenic lines as compared to control in two-tailed student’s T-test. *N* = 4.

Tuber yield of Rpi-amr3i_5C (transformed with *Rpi-amr3*) was significantly higher as compared control line (Maris Piper) whereas the increase in yield for other two transgenic lines, i.e. Rpi-amr1D_15A and Rpi-amr1D_15B (transformed with *Rpi-amr1*) were not statistically significant (Figure 4a). The percentage increase in yield for each transgenic line was 34% for Rpi-amr3_5C, as compared to Maris piper. The number of tubers for each genotype was also determined, and the results showed no statistically significant difference in the tuber count between the transgenic lines (Rpi-amr3_5C, Rpi-amr1D_15A, Rpi-amr1D_15B) and the Maris Piper (Figure 4b).

## Discussion

The present study aimed to evaluate late blight resistance in potato lines containing *S. americanum* resistance genes, specifically *Rpi-amr1* and *Rpi-amr3*, while also assessing yield and tuber count. Incorporating these resistance genes into *S. tuberosum* offers a promising strategy to manage late blight in potatoes, addressing food security and reducing dependency on chemical fungicides.^15,34,35^ The field trials in the study were conducted in Sweden, at a location with a complex natural population of *P. infestans*.

The field resistance to *P. infestans* was assessed through three consecutive years of trials, with disease progression quantified using the area under the disease progression curve (AUDPC). This method is widely used to evaluate resistance to polycyclic diseases like late blight.^24,25^ Visual disease scoring was conducted weekly by evaluating the percentage of infected foliage. Foliar infection in 2018, 2019, and 2020 was significantly lower in the three transgenic lines (*Rpi-amr3*, *Rpi-amr1D_15A*, and *Rpi-amr1D_15B*) compared to the Maris Piper, providing field resistance against diverse *P. infestans* populations. No prior field research has been published with late blight resistance conferred by the *Rpi-amr1* gene.

Our findings align with the results of previous field experiments conducted at Norwich Research Park, UK, in 2017 and 2018, where *Rpi-amr3* was tested for late blight resistance potato lines. In 2017, the plants were subjected to both natural and artificial inoculations, while in 2018, only artificial inoculations were applied due to the absence of natural infections.^18^ In general, the disease levels were lower in Sweden. The SSR genotyping of *P. infestans* samples from Southern Sweden revealed a dominance of new genotypes not recorded earlier in annual surveys. The only earlier known genotype found was EU_41_A2. This genotype has proven to be able to highly aggressive and be able to establish and dominate in different pathogen populations.^26^ However, a comparative study was not able to identify any specific traits that could explain the high competitiveness of EU_41_A2,^27^ with the possible exception of virulence. Determination of the virulence profiles based on Black’s differential set of 11 potato cultivars^28^ indicated that EU_41_A2 was able to overcome more R-genes compared to samples classified as “Other.” In 2020, a larger proportion of the *P. infestans* samples was identified as EU_41_A2 compared to 2018 and 2019, but no statical difference in AUDPC was found between the years.

Our results are also consistent with findings from Southern Sweden, where no late blight symptoms were observed in *S. nigrum* accessions, a close relative of *S. americanum*.^20^ The success of our field trials in Sweden underscores the genetic potential of *S. americanum* resistance genes and new genomic technologies (NGT) in potato. However, one step that is probably lacking for make use ofthese genes within the EU, is to create breeding material that include these genes within in potato breeder´s gene pool, since today you cannot directly cross *S. americanum* and *S. tuberosum*. In Europe, New Genomic Techniques (NGT) including cisgenesis (defined by only addition of genes from the breeder’s gene pool) have been brought forward as an important tool in achieving a sustainable crop production.^5,29,34^

Given the promising results, we recommend stacking different *R* genes, even those providing relative broad-spectrum resistance like *Rpi-amr3*, to strengthen protection against diverse and evolving *Phytophthora* populations. This recommendation is unpinned by our earlier results from the same field in Sweden (with two overlapping years) that gave even better results than the single genes from *S. americanum* used here. We did not detect any symptom development in King Edward potato plants carrying three different resistance genes (*Rpi-vnt1, Rpi-blb1* and *Rpi-blb2)*.^7^ The pyramiding strategy could offer a more sustainable and durable form of resistance, contributing to the long-term management of late blight, especially if several different stacks with 3 or more R genes could be used and recycled.
